# Smoking and diabetes: dangerous liaisons and confusing relationships

**DOI:** 10.1186/s13098-019-0482-2

**Published:** 2019-10-24

**Authors:** D. Campagna, A. Alamo, A. Di Pino, C. Russo, A. E. Calogero, F. Purrello, R. Polosa

**Affiliations:** 1 Centro per la Prevenzione e Cura del Tabagismo (CPCT), University Teaching Hospital “Policlinico-Vittorio Emanuele”, University of Catania, Catania, Italy; 2 U.O.C. MCAU, University Teaching Hospital “Policlinico-Vittorio Emanuele”, University of Catania, Catania, Italy; 3 Division of Andrology and Endocrinology, University Teaching Hospital “Policlinico-Vittorio Emanuele”, University of Catania, Catania, Italy; 4 Department of Clinical and Experimental Medicine, (MEDCLIN), University of Catania, Catania, Italy; 5 Center of Excellence for the Acceleration of HArm Reduction (CoEHAR), University of Catania, Catania, Italy

**Keywords:** Smoking, Diabetes, Cardiovascular complications, Smoking cessation

## Abstract

The combined harmful effects of cigarette smoking and hyperglycemia can accelerate vascular damage in patients with diabetes who smoke, as is well known. Can smoking cause diabetes? What are the effects of smoking on macro and microvascular complications? Now growing evidence indicates that regular smokers are at risk of developing incident diabetes. Since the prevalence rates of smoking in patients with diabetes are relatively similar to those of the general population, it is essential to address the main modifiable risk factor of smoking to prevent the onset of diabetes and delay the development of its complications. Quitting smoking shows clear benefits in terms of reducing or slowing the risk of cardiovascular morbidity and mortality in people with diabetes. Does quitting smoking decrease the incidence of diabetes and its progression? What are the effects of quitting smoking on complications? The current evidence does not seem to unequivocally suggest a positive role for quitting in patients with diabetes. Quitting smoking has also been shown to have a negative impact on body weight, glycemic control and subsequent increased risk of new-onset diabetes. Moreover, its role on microvascular complications of the disease is unclear. What are the current smoking cessation treatments, and which ones are better for patients with diabetes? Stopping smoking may be of value for diabetes prevention and management of the disease and its macrovascular and microvascular complications. Unfortunately, achieving long-lasting abstinence is not easy and novel approaches for managing these patients are needed. This narrative review examines the evidence on the impact of smoking and smoking cessation in patients with diabetes and particularly in type 2 diabetes mellitus and its complications. In addition, management options and potential future directions will be discussed.

## Background

Smoking as a mode of consumption is most commonly used for tobacco, mainly in the form of burnt tobacco and predominately cigarettes. Although the rate of cigarette smoking is decreasing in several countries, it remains a serious threat to public health worldwide, particularly in central and south-east Asia as well as in eastern Europe with the world’s largest number of smokers [1, 2]. The World Health Organization (WHO) estimates that by 2050 there will be one and a half billion smokers globally [3]. The devastating negative impact of cigarette smoking on health is well known, causing a wide range of diseases and disorders throughout every organ and system in the human body [4, 5]. The risks of developing cardiovascular diseases, cancer and chronic obstructive pulmonary diseases (COPD) are strongly correlated with the amount of daily consumption of cigarettes and the overall duration of smoking; prolonged smoking avoidance decreases these risks [6–8].

In addition to the smoking epidemic, another devastating pandemic looms: diabetes mellitus (DM). Since 1980, the number of adults with DM worldwide has quadrupled, exceeding 400 million people with a prognosis of nearly 650 million in the year 2040 (IFD Atlas 2015, 7th Edition, http://www.diabetesatlas.org) [9, 10]. The dramatic increase of DM prevalence represents a formidable challenge to public health. DM is characterized by a chronic hyperglycemia that causes irreversible damage to the blood vessels and consequently leading to macrovascular (coronary artery disease, stroke, peripheral arteriopathy and erectile dysfunction) and microvascular (retinopathy, nephropathy and diabetic neuropathy) complications of the disease [11]. Public health policies and programs must address the main modifiable risk factors for DM to prevent its onset and delay the development of its complications.

Cigarette smoking is one of the most important modifiable risk factor for DM [12]. Exposure to cigarette smoke is associated with vascular damage, endothelial dysfunction and activation of the blood-clotting cascade [13], so it is not at all surprising that the combined harmful effects of elevated blood glucose with cigarette smoking accelerates vascular damage in people with diabetes who smoke. It is widely accepted that cigarette smoking substantially increases the risk of micro and macrovascular complications in patients with type 2 DM (T2DM) [14–18].Quitting smoking substantially reduces this risk [17–20]. Even as reducing exposure to cigarette smoke is an imperative for public health, it is even more so for patients with DM, as reflected in most clinical guidelines [21].

Despite the increased risk resulting from the combination of chronic hyperglycemia and regular exposure to cigarette smoking, the prevalence of smoking among people with DM appears to be similar to that of the general population [22]. In the United States, the prevalence of tobacco consumption has decreased significantly, but this positive trend has not occurred among patients with DM [23]. Therefore, tackling cigarette smoking in patients with diabetes requires greater effort and the investment of additional resources to implement more targeted and intensive anti-smoking strategies.

After selecting published key studies, we have examined the evidence on the impact of smoking and smoking cessation on DM and its complications, particularly in patients with T2DM. In addition, management options for these patients and potential future directions will be discussed.

## Can smoking cause diabetes?

Evidence supporting an increased risk for T2DM in smokers has been accumulating for over 20 years. In 1997, Kawakami et al. investigated the effects of smoking on the incidence of non-insulin-dependent diabetes mellitus (NIDDM) in a cohort of 2312 Japanese males. After controlling for other known risk factors for NIDDM, a proportional hazards regression analysis indicated that those who were currently smoking 16–25 cigarettes per day had a 3.27 times higher risk of developing NIDDM during the follow-up period than never smokers [24]. In 2007, a first meta-analysis of 25 prospective cohort studies showed a dose-dependent association between smoking and incident T2DM [25]; the relative risk (RR) for incident disease of 1.61 in smokers of ≥ 20 cigarettes per day compared to non-smokers, decreases to 1.29 and 1.23 in smokers of < 20 cigarettes a day and former-smokers, respectively. In 2015, a second meta-analysis of 88 prospective cohort studies (almost 300,000 cases of new-onset T2DM), confirmed a significant association between smoking and T2DM risk, with a RR of 1.37 in smokers and 1.14 in former smokers compared to never smokers [12]. Moreover, a clear dose–response relationship was demonstrated in the analyses with the level of cumulative exposure to cigarette smoke over time (i.e. pack/years). The authors estimated that at least 25 million cases of T2DM worldwide could be directly attributable to cigarette smoking alone.

Subsequently, the association between smoking and diabetes has been also reported in studies of Asiatic populations [26–28]. In 2017, a meta-analysis of 22 prospective studies in Japan (16,383 patients with T2DM) showed similar associations, with the pooled RR of T2DM of 1.38 for current smokers and 1.19 for former smokers as compared with never smokers [29]. The authors estimated that 18.8% of T2DM cases in men and 5.4% of T2DM cases in women were attributable to smoking. While the risk of T2DM remained elevated among those who quit smoking within the preceding 5 years, the risk decreased steadily thereafter, declining after 10 years of cessation to a risk level comparable to that of never smokers.

Despite this evidence of an association between cigarette smoking and increased risk of T2DM, a cause-effect link between smoking and T2DM cannot be established with certainty because other risk factors play a role, such as stress, diet, levels of physical activity and distribution of body fat. To uncover the real impact of smoking on the onset of DM, these well-known confounding risk factors must be bracketed out or adjusted through analysis.

The relationship between cumulative smoking exposure and pre-diabetes [30] in a cross-sectional study of 2142 healthy Europeans (25 to 41 years old) showed an increased risk of pre-diabetes in smokers compared to non-smokers (OR = 1.82). As in the T2DM review, the researchers demonstrated a dose–response association with progressively increasing ORs in smokers with pre-diabetes, from an OR of 1.34 in smokers with exposure < 5 pack-years, to 1.80 for 5 to 10 pack-years, and 2.51 for > 10 pack-years. A similar demonstration of a significantly increased risk of pre-diabetes for smokers has been also reported in a study from New Mexico [31]. These findings point to a mechanistic hypothesis that smoking accelerates progression from normoglycaemia to impaired glucose tolerance status possibly by eliciting development of insulin resistance, increasing the risk of developing diabetes in smokers. However, the risk of smoking associated with pre-diabetes appears to be much higher [32] than that for DM as presented in the meta-analysis; this difference may be the result of small sample size and cross-sectional design of the studies conducted on pre-diabetes.

Smoking is a dangerous liaison that may lead to pre-diabetes and DM. Although there is strong evidence that smoking is associated with development of diabetes, more work is needed to provide confirmation about possible causal links.

## Does quitting smoking decrease the incidence of diabetes?

Given the evidence indicating that regular smokers are at risk of developing diabetes, abstinence from smoking should lower diabetes prevalence. However, this does not seem to be the case. On the contrary, evidence shows that quitting smoking may increase the risk of new-onset T2DM, even if this risk tends to decrease progressively over the long term. A systematic review of 10 prospective cohort studies evaluated the impact of smoking cessation on the risk of T2DM and found a significant increase in risk (RR = 1.54) for those who had quit smoking for less than 5 years compared to never smokers. However, this risk decreased progressively in those who stopped smoking for longer than 5 years; the RR declined to 1.18 for those abstaining 5–9 years, and 1.11 for ≥ 10 years [12]. Of note, subgroup analysis showed that the increase in short-term risk was more evident in Asians than in European or North American populations [12].

These findings are in line with the results of a study conducted on 53,930 Japanese employees of the Japan Epidemiology Collaboration on Occupational Health Study [26]. The risk of incident diabetes (RR = 1.36) in ex-smokers who had quit for less than 5 years declined gradually to RR 1.23 in those who had stopped smoking between 5 and 9 years and declined to an almost equal risk of a non-smoker of RR 1.02 for those abstinent for over 10 years (RR = 1.02). In a retrospective study conducted on 8452 male T2DM patients in Taiwan [33], the RR was was 1.50 for smokers compared to non-smokers. Smoking abstinence contributed to increasing the risk for T2DM in the first year after quitting smoking (RR = 1.83) and in the second year (RR = 2.02), declining rapidly thereafter.

Why is there an increased risk of incident T2DM after stopping smoking?. One possibility is that it may result from the overall cumulative exposure to smoking before quitting as shown in studies that have reported higher level of risk in heavy inveterate smokers compared to light occasional smokers and that have compared this risk between Asians and Europeans or Americans, known to be very high in the former [34–36]. Another possibility is the weight gain and the increase in waist circumference that occurs with quitting influencing the development of insulin resistance—this would also explain the increased risk of developing diabetes observed after quitting. A recent analysis conducted on three cohort studies found that the risk of developing T2DM was proportional to body weight gain after smoking cessation, and increased risk was not observed in those who did not gain weight [37]. Post-cessation weight gain could be a plausible hypothesis for the increased risk of developing T2DM.

The studies display a confusing and counterintuitive relationship between cessation and an increase in the medium-term risk for developing diabetes. Nevertheless, the value of smoking abstinence for diabetes prevention and management cannot be discounted and should be always communicated by healthcare providers to all their patients who smoke, especially those with diabetes [38, 39]., Because post-cessation weight gain is a risk factor, those who are quitting smoking should be counseled to on ways to limit their weight gain, and not accept weight gain as an unavoidable side-effect of cessation.

## Smoking and vascular complications

### Macrovascular complications

Macrovascular complications, including ischemic heart disease, stroke and peripheral arterial disease, are the main causes of morbidity and mortality in patients with DM, with a risk of cardiovascular events up to 4 times greater than in the general population [40, 41]. Smoking is one of the key risk factors for cardiovascular disease (CDV), which contributes substantially to the overall cardiovascular burden [42, 43]. Consequently smoking increases the risk of macrovascular complications in patients with DM.

The relationship between smoking, DM and cardiovascular risk has been investigated in several studies. Pan and colleagues [20] performed a systematic review and meta-analysis of prospective cohort studies on patients with diabetes who smoked regularly and the risk of total mortality and cardiovascular events. The review includes 48 studies on smoking and risk of total mortality, 13 on cardiovascular mortality, 16 on total cardiovascular disease, 21 on coronary heart disease, 15 on stroke, 3 on peripheral artery disease and 4 on heart failure. The analyses pooled an adjusted RR associated with smoking of 1.55 for total mortality and 1.49 for cardiovascular mortality. For patients with diabetes, smoking increased the pooled RR for total cardiovascular disease as 1.44, coronary heart disease (CHD) as 1.51, stroke as 1.54 and heart failure as 1.43. It is noteworthy that the risk of peripheral arterial disease was more than double in patients with diabetes who smoke at RR = 2.15.

People with diabetes who had stopped smoking still exhibited an elevated risk of about 10–20% of total mortality, cardiovascular mortality, total CVD and CHD compared with never smokers, with the only exception of stroke. These findings concur with the results of an earlier meta-analysis on 46 studies that showed a higher risk of total mortality as well as cardiovascular outcomes, and for CHD than other events in patients with diabetes [19]. Moreover, a decreasing trend was observed in smoking quitters [19].

Recently three studies have corroborated these findings. In one, a cohort study conducted on a large population from the Swedish National Diabetes Register, smoking was one of the five strongest predictors of death and acute myocardial infarction among patients with T2DM; (the other predictors are glycated hemoglobin, systolic blood pressure, LDL cholesterol, and physical activity) [43]. In another retrospective study on a population-based cohort of 132,462 Chinese patients (receiving public primary care services during 2010) determined that the smoking habit was associated with increased risk of all causes of mortality in men at RR = 1.71 and in women at RR = 2.04 [44]. In the third study, a large prospective cohort study assessed the risk of CHD incidence and mortality, and all-cause mortality in Finnish people with and without T2DM according to smoking status [45]. The study showed that smokers with T2DM had an increased CHD mortality risk of HR 6.15 for men; HR 6.92 for women compared to T2DM nonsmokers at HR 2.62 for men and HR 4.06 for women. The risk of CHD in T2DM patients who had stopped smoking was still significantly higher than in their non-smoking non-diabetic counterparts, but lower than in T2DM patients who still smoked. Similar results were observed for all-cause mortality data [45]. These studies make it clear that the risk for macrovascular complications is higher for patients with diabetes who smoke than those who do not.

### Microvascular complications

As opposed to the quantity of research available on macrovascular complication, few studies have examined the relationship between smoking and microvascular complications such as nephropathy, retinopathy and neuropathy. The results of the studies are not entirely consistent, in particular for T2DM.

#### Nephropathy

Diabetic nephropathy is a clinical syndrome characterized by persistent albuminuria, progressive decline in the glomerular filtration rate (GFR), peripheral edema, and elevated arterial blood pressure. It is one of the most severe complications in patients with diabetes, and it is considered a major cause of end-stage renal failure [46, 47]. There is accumulating evidence that smoking increases the risk of incidence and progression of nephropathy in people with diabetes, and particularly in those with T1DM [48–51].

In a 4-year prospective study of 943 T1DM patients with normoalbuminuria at baseline, Scott et al. [52], reported the development of persistent albuminuria in 109 of the 943 subjects. Current smoking had a substantial potentiating effect, as well as poor glycemic control (HbA1C > 8%), for the onset of persistent albuminuria.

Feodoroff and colleagues explored the effect of smoking on development and progression of diabetic nephropathy (expressed as 12-year cumulative risk of microalbuminuria, macroalbuminuria and end-stage renal disease) in a large cohort of patients with T1DM from a prospective Finnish Diabetic Nephropathy study [53]. The authors reported active smoking as a risk factor for progression of diabetic nephropathy with a dose-dependent risk increase. For those who quit smoking their risk for the development and progression of diabetic nephropathy was the same as for nonsmokers after multivariable adjustment [53].

Other evidence on the association between smoking and diabetic nephropathy in T2DM is inconclusive. For example, the higher risk of having a low glomerular filtrate rate compared with non-smokers (OR = 2.20) was only significant in male patients [54]. A similar gender-dependent association was reported by Briganti and colleagues for male patients with high-normal systolic blood pressure or with high-normal 2-h glucose levels [55]. A more rapid progression of diabetic nephropathy has been observed more frequently in smokers with T2DM compared to non-smoking patients [56–59]. A study conducted on Taiwanese men showed a clear dose–response effect of cigarette smoking on the development of proteinuria in males with T2DM [60]. Compared with non-smokers, those who had smoked 15–30 or more than 30 pack-years were respectively 2.78 and 3.20 times more likely to develop proteinuria. The dose–response effect of tobacco exposure on the development of proteinuria was highly significant in all patients, including subgroups with a relatively short duration of DM, optimal blood pressure control, and those of young age. Progression of microalbuminuria to overt proteinuria and subsequent terminal renal failure were higher in smokers than in non-smokers. A recent meta-analysis of 21 studies assessed the impact of smoking on diabetic nephropathy and reported that smoking was a statistically significant risk factor for diabetic nephropathy with an OR of 1.7 [48]. A recent meta-analysis of 20,056 patients with T2DM found that the odds ratio (OR) of smokers developing albuminuria compared to non-smokers was 2.13 (95% CI 1.32, 3.45) with the only other statistically significant enhancer of risk being the duration of disease [61].

#### Retinopathy

The role of smoking as a potential risk factor for diabetic retinopathy has been established in patients with type 1 DM [62, 63], but its role is disputed in patients with T2DM, with many studies reporting no association or even a decreased risk of developing retinopathy in smokers [64–69]. In the 4-year and 10-year follow-up of the Wisconsin Epidemiologic Study of Diabetic Retinopathy, smoking was not significantly associated with the risk of incidence and progression of diabetic retinopathy [67]. Data from the United Kingdom Prospective Diabetes Study (UKPDS) showed that retinopathy onset (incidence) in the 6-year follow-up of 1216 patients with T2DM was not associated with smoking [64]. In the study, the progression of vasculopathy was much less rapid in smokers compared with non-smokers among the 703 patients who had diabetic retinopathy at the beginning of the study. This discordant evidence that—compared to non-smokers—the risk of diabetic retinopathy is significantly increased in smokers with T1DM while significantly decreased in smokers with T2DM has been also confirmed recently in a meta-analysis of 73 studies [70].

#### Neuropathy

The association between smoking and the risk of diabetic neuropathy has been examined in two important articles. In the European Diabetes Prospective Complications Study, neuropathy was assessed at baseline and after a 7.3-year follow-up in 1172 patients with type 1 DM from (31 centers), the study showed that, apart with glycemic control, the incidence of neuropathy was significantly associated with smoking at OR = 1.68 [71]. The second article, a systematic review of 10 prospective cohort and 28 cross-sectional studies [72], evaluated the development of diabetic neuropathy in a total population of 5558 patients. Over a period of 2 to 10 years, 1550 new cases of diabetic neuropathy were observed; the OR for neuropathy among smokers was not significantly higher. In a secondary analysis, authors found a significant association of smoking with diabetic neuropathy in patients with type 1 (OR = 1.74; 7 studies), but not for patients with type 2 DM (OR = 0.65; 3 studies) [72].

These discrepancies could be the result of the poor sensitivity of common methods of neuropathy testing [73, 74]. Of interest, Ahmad and colleagues [75], by using more sensitive and specific nerve conduction studies, were able to show that smoking was an independent risk factor for manifestations of neuropathy in patients with T2DM, with heavy smokers exhibiting worse nerve conduction.

The studies on smoking and its effects on microvascular effects can seem to present a confusing relationship until other factors are considered. The impact of smoking on these conditions varies by the type of diabetes, DM or T2DM, and by gender as well. Overall, very few rigorous prospective studies are available, and, as is too often the case, more research is necessary.

## Smoking and glycemic control

The effect of smoking on glycemic control in people with diabetes is poorly studied with often contradictory results. Cigarette smoking worsens insulin-resistance in patients with diabetes [76]; consequently, quitting smoking should improve glycemic control. Yet, smoking cessation often results in worsened glycaemic control, possibly due to the weight gain that frequently occurs after smoking abstinence [77].

A Japanese study of 25 patients with diabetes who smoke indicated poorer glycemic control in those who quit compared to patients who continued to smoke [78]. The English cohort study THIN (The Health Improvement Network) also showed an association between quitting smoking and worse glycemic control in T2DM patients [79, 80]. The effects of continued smoking in the data from the Fukuoka Diabetes Registry [81] and the Swedish National Diabetes Registry [82] showed that HbA1c levels progressively increased with the number of cigarettes smoked per day. Notwithstanding other studies have not confirmed any association between smoking and glycemic control [76, 83].

In another cohort study of 10,551 men and 15,297 Chinese women with DM, smoking was associated with an increased risk OR of 1.49 in men and 1.56 in women for poor glycemic control (defined as HbA1c ≥ 7.0%), particularly in elderly patients [84]. The relationship is dose-dependent and independent of traditional confounding factors, including sociodemographic and lifestyle factors. The increased risk for poor glycemic control compared to non-smokers normalized only after at least 10 years of abstinence from smoking. Another study conducted in China [85] on 7763 male patients with T2DM found that cigarette smoking was associated with increased level in fasting plasma glucose and HbA1c, particularly in treated patients with highest smoking duration and pack-years. Compared to non-smokers, average HbA1c increase was 0.27% in current smokers with a smoking duration of ≥ 30 years and 0.38% for smoking ≥ 40 pack-years. These inconsistent results could be explained by the differences in the study populations. The discrepancies may be caused by confounding factors, in particular, known lifestyle risk factors that were not examined in some of the studies.

## Impact of quitting smoking on diabetes complications

Quitting smoking, shows clear benefits in terms of reduction or slowing of the risk for cardiovascular morbidity and mortality in people with diabetes as it does for the general population [86, 87]. The large meta-analysis by Pan and colleagues [20] discussed earlier has shown that patients who quit smoking have a lower cardiovascular risk compared to smokers. In T2DM patients, smoking cessation is known to decrease both short- and long-term CVD risk, even independently from weight gain [88, 89]. In addition, data from 11,140 patients with T2DM in the ADVANCE study have shown that quitting smoking was associated with a 30% decrease in all-cause mortality and the benefits for reducing cardiovascular events were generally more consistent in patients who had stopped smoking for more than 10 years compared to those who had only recently stopped [90]. More recently, a descriptive analysis of 890 Spanish patients with T2DM (444 smokers and 446 former-smokers) performed in a cross-sectional, multicenter, nationwide study, assessed the estimated likelihood of CHD risk at 10 years (according to the UKPDS score) in 890 patients with diabetes [91]. The estimated risk of developing CHD was significantly greater in smokers compared with former-smokers. And specifically, in those subjects with poorer glycemic control (HbA1c > 7%) compared to those with adequate glycaemic control (HbA1c ≤ 7%). The promising finding from a nephropathy study demonstrated the quitting smoking reduced the risk of that complication to that of a never smoker. Still for evidence on microvascular complications, the studies are limited and not conclusive. For instance, two studies have shown that smoking cessation among patients with diabetic nephropathy improved the progression of existing nephropathy [18, 59], but its impact on newly developing diabetic nephropathy has been infrequently studied with prospective research designs.

The evidence supporting the position that quitting smoking can lower the risk of macrovascular complications among patients with diabetes is sound. We can be sure that quitting can break-up that dangerous liaison. On the other hand, the impact of smoking cessation on the risk of microvascular complications remains without clarity, a set of confusing relationships. Further prospective studies will be needed to document and quantify the decreasing of risk of complications in patients with diabetes who stop smoking.

## Smoking cessation for people with diabetes

Abstinence from smoking will certainly produce specific benefits in patients with diabetes. This fact is reflected in the most recent guidelines on diabetes treatment [21, 92] which include smoking cessation as a key chapter. Current guidance highlights the importance of stopping smoking for patients with diabetes to achieve a better quality of life and to delay the onset and progression of diabetes complications.

The currently available smoking cessation therapies have been shown to double or even triple the dropout rates in controlled studies [93, 94]. A recent study in patients with DM yielded a smoking cessation rate of 11.1% at 6-months in those undergoing an intensive smoking cessation program [95]. However, according to a survey by Diabetes UK, 64.1% of smokers with DM (64.1%) do not receive any assistance or advice to quit [96]—far too many. Another constraint to cessation treatment is the absence of a convincing demonstration of an effective cessation interventions in patients with DM [97]. Further studies will be needed to provide clear evidence that which interventions can be valuable for these patients. As a consequence the smoking prevalence among patients with DM continues to be similar to that found in the general population with a significantly less marked decrease trend in patients with diabetes compared to the general population [22, 23, 98, 99]. These conditions mean that helping patients with diabetes to quit requires a greater commitment and the use of personalized anti-smoking strategies.

Given the high risk for relapse, successful and prolonged smoking abstinence can be challenging. Psychological support appears to be a central component of treatment. Combining personalized psychological support with standard pharmacological medications can achieve the best possible results [100, 101]. A recent Cochrane review showed that personalized counseling of > 10 min significantly increases the likelihood of quitting up by 40 to60% [102]. Patients with diabetes who smoke should be routinely reminded that cigarette smoking increases their risk of developing disease complications, adversely affects their blood glucose control and increases their insulin-resistance. For treatment, the first line drugs used to increase the likelihood of success in smoking cessation include nicotine replacement therapy (NRT), bupropion and varenicline [100, 101], discussed below.

NRT is available in different formulations: chewing gum, inhalers, lozenges, sprays and transdermal patches. Their main mechanism of action is that of replacing the nicotine delivered by cigarette smoking, thus decreasing the severity of withdrawal symptoms and helping the smoker to quit [100]. Different formulations may have a distinct impact on withdrawal symptoms or on the urge to smoke, but whether one formulation is more effective than another is open to debate. Nonetheless, NRT-based treatment doubles the chances of success in quitting smoking, regardless of the specific formulation [102–104]. Although not formally regulated as a pharmaceutical product, electronic cigarettes are nicotinic substitutes. They are battery-powered devices that vaporize the nicotine present in the refill liquid of electronic cigarettes and, like NRT, are able to lower the severity of withdrawal symptoms [105, 106]. Randomized clinical trials support the efficacy and safety of these devices [107–109]. In particular, a recent RCT has demonstrated that electronic cigarettes are on average twice as effective as NRT for smoking cessation [109]. Similar positive findings have been recently published; in a large pragmatic RCT of 1124 smokers, 7% participants quit smoking in the NRT patches plus nicotine e-cigarette group compared with 2% in the patches only group [110]. Nicotinic substitutes—by virtue of the known effects on sympathetic neural stimulation and catecholamine release—can have a negative impact on the cardiovascular system and on glucose metabolism [111, 112]. Some authors have raised concerns about NRTs use in DM patients with poor glyco-metabolic control given that nicotine may increase insulin-resistance [112, 113]. Therefore, clinicians must consider the possibility of clinical-metabolic worsening of DM and its complications during NRT therapy. Some studies have shown an association between the use of NRT and reporting of serious cardiovascular events (e.g. myocardial infarction), but these events were primarily reported for patients who continued to smoke while using NRT [114]. Two meta-analyses investigating adverse events associated with NRTs have shown increased cardiovascular symptoms (including tachycardia and chest pain) [115, 116], but not major cardiovascular events (cardiovascular death, non-fatal myocardial infarction and non-fatal stroke) [116]. A cohort study of 50,214 smokers who tried to quit smoking [117] with 4-weeks use of NRT did not find any impact on cardiovascular risk. Although no specific recommendations for smokers with DM are available, it is reasonable to limit the use of NRT over time.

Buproprion was initially developed and marketed as an antidepressant, but it has become the first oral treatment without nicotine approved for smoking cessation. It inhibits the re-uptake of norepinephrine and dopamine at the level of neuronal synapses in the central nervous system, acting as a non-competitive antagonist of nicotine receptors. In a Cochrane review, bupropion doubles the odds of quitting smoking compared to placebo, with or without co-occurring depression [118]. The cessation rates for bupropion treatment are practically similar to those obtained with NRT [118]. Bupropion was determined to be safe in patients with cardiovascular disease, although occasional increases in blood pressure have been reported in smokers with hypertension [119]. Although no studies are available in patients with DM, the use of bupropion can be considered safe for these patients. A plus for this treatment is that bupropion is able to limit the weight gain that often occurs when smoking is stopped, as demonstrated in RCTs [120, 121]. Bupropion could therefore be proposed as a treatment of choice in obese patients with diabetes.

Another cessation treatment is varenicline. It is a selective partial agonist of the α4ß2 nicotinic acetylcholine receptors in the ventral tegmental area of the brain which acts by attenuating the withdrawal symptoms that arise when quitting smoking [122, 123]. Many RCTs have confirmed the efficacy of varenicline. A Cochrane review concluded that varenicline more than doubles the odds of quitting smoking compared to placebo [124]. Furthermore, varenicline showed its greater efficacy compared to any form of bupropion monotherapy or with NRT [124, 125]. When compared to NRT combination therapy, varenicline significantly increases the success rate in the short and medium term, but not in the long term [126, 127]. The results raise questions about the relative effectiveness of intense smoking pharmacotherapies. The safety profile, varenecline appears to be safe and well tolerated by patients with DM. A retrospective analysis of data obtained from participants in 15 randomized clinical trials with varenecline showed that the distribution of the number of adverse events in patients with DM (mainly nausea and headache) was comparable to that of participants without diabetes [128]. In a test of varenicline’s effectiveness, our research group has recently completed the first randomized, double-blind, placebo-controlled study on the efficacy and safety of varenicline in smokers with T2DM and demonstrated a quit rate of 22% at 52-weeks [129].

## Conclusions and future directions

The complex interaction between smoking and DM poses multiple challenges for the researcher, the clinician and the patient. Current evidence shows that regular smoking is an important risk factor for cardiovascular morbidity and mortality in patients with diabetes. Although the role of smoking and the impact of smoking cessation on microvascular complications has not been fully clarified, stopping smoking must remain a primary goal for people with diabetes to decrease their risk for macrovascular complications.

The patient with diabetes who smokes may represent a distinct ‘‘phenotype’’ with different glyco-metabolic profile, accelerated vascular damage, variable progression of macro and micro-vascular complications and poor response to smoking cessation management. Given that not all smokers with DM are susceptible to the detrimental effects of cigarette smoke, searching key phenotypic predictors for this vulnerability may be an important area for future investigation.

The increased recognition that regular smoking and DM is a dangerous liaison albeit with confusing relationships should stimulate greater efforts to develop effective smoking cessation programs and encourage avoidance strategies. The high smoking prevalence among patients with diabetes, their poor level of glyco-metabolic control and their low success rates of stopping smoking all highlight the importance of systematically counseling smokers with DM of the numerous risks of smoking.

Doctors and healthcare providers therefore have a duty to alert their patients with diabetes about the additional burden of risks of caused by smoking. The message must be strong and personalized. Physicians should evaluate the need to prescribe drugs for the treatment of nicotine addiction to decrease nicotine withdrawal symptoms that may occur: dysphoric or depressed mood, irritability, frustration or anger, anxiety and restlessness, increased cough, increased appetite, weight gain, sense of weakness and constipation. Physicians should not hesitate to refer these patients to a specialized center and follow-up on their course of treatment.

Alas, the solid bond with cigarette smoking creates a huge obstacle for the smoker, even for those who have a strong desire to quit, so much so that several attempts and treatments must be attempted before obtaining a lasting abstinence. Where success has not be achieved, clinicians should consider alternative strategies including those based on risk reduction by using the new emerging technological devices without combustion (e.g. electronic cigarettes and heated tobacco devices) [130, 131]. Although, little is known about the health effects of long-term vaping or heated tobacco systems, we know for sure that long-term consumption of combustible cigarette is deadly liasion and can lead to the development of diabetes and other metabolic alterations. The recent outbreak of severe acute respiratory illnesses among several hundred US young adults and teens is NOT linked to commercial nicotine vaping products; the evidence is mounting that the actual source of these illnesses is the consumption of some illegal, black market THC carts (cartridges) containing dangerous adulterants as recently stated by the FDA [132].

Given that many patients with diabetes continue smoking despite the well-known health risks, these emerging technologies for nicotine delivery could be a viable and much less harmful alternative. We are aware of only one paper investigating the impact of e-cigarette use in diabetes. A large Internet-based survey of 574 regular e-cigarette users with diabetes [133] found that 41.9% of the respondents reported improvement in diabetes control after switching away conventional cigarettes to e-cigarettes, whereas worsening was reported in only 0.6%. More studies in smokers with diabetes will be required to confirm these initial findings.

Smoking and diabetes presents both a dangerous liaison and confusing relationships. While we wait for further research for more evidence, promoting smoking cessation for those with DM deserves to be a top priority. Encouraging all smokers to quit may reduce the number of cases of DM overall.

## Data Availability

Not applicable.

## Abbreviations

WHO: World Health Organization
COPD: chronic obstructive pulmonary diseases
DM: diabetes mellitus
T2DM: diabetes mellitus type 2
RR: relative risk
CVD: cardiovascular disease
CHD: coronary heart disease
NRT: nicotine replacement therapy
