# Correction: Smoking and diabetes: dangerous liaisons and confusing relationships

**DOI:** 10.1186/s13098-023-01099-6

**Published:** 2023-06-02

**Authors:** D. Campagna, A. Alamo, A. Di Pino, C. Russo, A. E. Calogero, F. Purrello, R. Polosa

**Affiliations:** 1 Centro per la Prevenzione e Cura del Tabagismo (CPCT), University Teaching Hospital “Policlinico-Vittorio Emanuele”, University of Catania, Catania, Italy; 2 U.O.C. MCAU, University Teaching Hospital “Policlinico-Vittorio Emanuele”, University of Catania, Catania, Italy; 3 Division of Andrology and Endocrinology, University Teaching Hospital “Policlinico-Vittorio Emanuele”, University of Catania, Catania, Italy; 4 Department of Clinical and Experimental Medicine, (MEDCLIN), University of Catania, Catania, Italy; 5 Center of Excellence for the Acceleration of HArm Reduction (CoEHAR), University of Catania, Catania, Italy

**Correction: Diabetol Metab Syndr (2019) 11:85** 10.1186/s13098-019-0482-2

Following publication of the original article [1], the authors would like to correct the conflict of interest statement as given below.

This paper was partly supported by grant WS5086648 from GRAND (Global Research Award for Nicotine Dependence), an independently reviewed competitive grants program funded by Pfizer Inc, a US biopharmaceutical company active in the research, production and marketing of smoking cessation drugs (https://www.pfizer.com). Support for publication fees and open access is provided by ECLAT srl, a research based spin-off company of the University of Catania, with the help of a grant from the Foundation for a Smoke-Free World Inc., a US nonprofit 501(c)(3) private foundation (https://www.smokefreeworld.org) which receives funding from Philip Morris. The contents, selection, and presentation of facts, as well as any opinions expressed herein are the sole responsibility of the authors and under no circumstances shall be regarded as reflecting the positions of the sponsors.
